# Restoring large-scale brain networks in PTSD and related disorders: a proposal for neuroscientifically-informed treatment interventions

**DOI:** 10.3402/ejpt.v6.27313

**Published:** 2015-03-31

**Authors:** Ruth A. Lanius, Paul A. Frewen, Mischa Tursich, Rakesh Jetly, Margaret C. McKinnon

**Affiliations:** 1 Western University, London, ON, Canada; 2 Lawson Health Research Institute, London, ON, Canada; 3 Canadian Forces, Health Services, Ottawa, Ontario, Canada; 4 McMaster University, Hamilton, Hamilton, Ontario, Canada; 5 St. Joseph's Healthcare Hamilton, Hamilton, Ontario, Canada; 6 Homewood Research Institute, Guelph, Ontario, Canada

**Keywords:** Intrinsic networks, default mode network, salience network, central executive network, insula, PTSD, interoception, neurofeedback, mindfulness, dissociation

## Abstract

**Background:**

Three intrinsic connectivity networks in the brain, namely the central executive, salience, and default mode networks, have been identified as crucial to the understanding of higher cognitive functioning, and the functioning of these networks has been suggested to be impaired in psychopathology, including posttraumatic stress disorder (PTSD).

**Objective:**

1) To describe three main large-scale networks of the human brain; 2) to discuss the functioning of these neural networks in PTSD and related symptoms; and 3) to offer hypotheses for neuroscientifically-informed interventions based on treating the abnormalities observed in these neural networks in PTSD and related disorders.

**Method:**

Literature relevant to this commentary was reviewed.

**Results:**

Increasing evidence for altered functioning of the central executive, salience, and default mode networks in PTSD has been demonstrated. We suggest that each network is associated with specific clinical symptoms observed in PTSD, including cognitive dysfunction (central executive network), increased and decreased arousal/interoception (salience network), and an altered sense of self (default mode network). Specific testable neuroscientifically-informed treatments aimed to restore each of these neural networks and related clinical dysfunction are proposed.

**Conclusions:**

Neuroscientifically-informed treatment interventions will be essential to future research agendas aimed at targeting specific PTSD and related symptoms.

The objective of this commentary is threefold: 1) to describe three main large-scale networks of the human brain; 2) to discuss the functioning of these neural networks in posttraumatic stress disorder (PTSD); and 3) to offer hypotheses for neuroscientifically-informed interventions based on treating the abnormalities observed in these neural networks in PTSD and related disorders.

Three intrinsic connectivity networks (ICN) in the brain have been identified as crucial to the understanding of higher cognitive function (Menon, 2011), namely the central executive (CEN), salience (SN), and default mode (DMN) networks. Responses of these ICNs are thought to generally increase and decrease proportionally and antagonistically during performance of cognitive and emotional-processing tasks. The CEN is a network that is crucial to verbal learning and executive functioning (Habas et al., 2009; Koechlin & Hyafil, 2007; Koechlin & Summerfield, 2007; Miller & Cohen, 2001; Petrides, 2005; Seeley et al., 2007). The SN consists of the dorsal anterior cingulate cortex and the frontoinsular cortex and plays a key role in salience detection, in other words, directing behavior to the most pertinent actions (Dosenbach et al., 2007; Lovero, Simmons, Aron, & Paulus, 2009; Seeley et al., 2007; Sridharan, Levitin, & Menon, 2008). The DMN, consisting of anterior and posterior medial cortices and lateral parietal lobes, plays an important role in self-referential processing, autobiographical memory, and social cognition (Amodio & Frith, 2006; Buckner, Andrews-Hanna, & Schacter, 2008; Greicius, Krasnow, Reiss, & Menon, 2003; Qin & Northoff, 2011; Raichle et al., 2001; Spreng, Mar, & Kim, 2009). Crucially, the anterior insula of the SN is thought to mediate the engagement of the CEN and disengagement of the DMN, and thus the dynamic interplay between externally- and internally-focused attention and cognitive-affective processing (Menon & Uddin, 2010; Seeley et al., 2007; Sridharan et al., 2008).

## Central executive network and PTSD

Several authors have examined CEN activity during cognitive processing in PTSD (Daniels et al., 2010; St. Jacques, Kragel, & Rubin, 2013). During a working memory task, whereas controls showed significantly stronger connectivity within areas implicated in the CEN, including the right inferior frontal gyrus and the right inferior parietal lobule, the PTSD group showed stronger connectivity with areas implicated in the DMN (namely enhanced connectivity between the medial prefrontal cortex and the left parahippocampal gyrus). Using an autobiographical memory retrieval paradigm, however, PTSD was associated, among other findings, with decreased recruitment of two networks identified as DMN-associated (medial temporal lobe and medial prefrontal cortex networks) when recalling autobiographical memories (St. Jacques et al., 2013). In addition, memories recalled in first-person perspective tended to recruit the medial temporal lobe network more so than those recalled in third-person. Finally, increased emotional intensity of the memory was associated with increased frontoparietal network activity for healthy controls but not for PTSD participants (St. Jacques et al., 2013).

Decreased resting-state connectivity within the CEN has also been found between PTSD participants and trauma-exposed controls during an emotional face viewing paradigm, with decreased connectivity between frontoparietal regions within a CEN component associated with trauma history and with PTSD symptoms (Cisler, Steele, Smitherman, Lenow, & Kilts, 2013). We also reported a positive correlation between frequency of dissociative experiences and DMN connectivity with the dorsolateral prefrontal cortex, a region involved in the CEN (Bluhm et al., 2009). This suggests that dissociative experiences may involve alterations in the relation between the DMN and CEN, which may relate to difficulties switching between DMN and CEN.

These differential patterns of connectivity are related to significant group differences with task-induced switches (i.e., engaging and disengaging the DMN and the CEN) (Daniels et al., 2010). Specifically, whereas controls engaged executive networks required for successful working memory performance, individuals with PTSD tended to engage brain regions involved in task-irrelevant processes (e.g., self-referential processing; connectivity between the posterior cingulate cortex (PCC) and the right superior frontal gyrus and between the medial prefrontal cortex and the left parahippocampal gyrus), signaling a potential key mechanism underlying the cognitive dysfunction observed in this population. By contrast, effective recruitment of brain regions associated with self-referential processing during autobiographical memory recall also seems to be impaired in PTSD. Taken together, these results point towards alterations in the ability to engage and switch between task-relevant (i.e., CEN) and task-irrelevant (i.e., DMN) brain networks during cognitive processing among individuals with PTSD, a function that is in turn known to be partly mediated by the anterior insula.

## Restoring the CEN through cognitive remediation

In addition to its core affective components, PTSD is associated with poor cognitive functioning across multiple domains, including declarative memory (Moradi, Abdi, Fathi-Ashtiani, Dalgleish, & Jobson, 2012), short-term memory (Johnsen & Asbjornsen, 2008), attention, and executive functioning (Aupperle, Melrose, Stein, & Paulus, 2012; Polak, Witteveen, Reitsma, & Olff, 2012). The presence of cognitive impairments, in particular, deficits in executive functioning and memory, has also been associated with poor functional outcomes (e.g., return to work) among patients with affective disorders (Altshuler et al., 2007; Altshuler, Bearden, Green, Van Gorp, & Mintz, 2008; Bowie et al., 2010; Depp et al., 2009; Dickerson et al., 2004; Geuze, Vermetten, De Kloet, Hijman, & Westenberg, 2009; Gildengers et al., 2007). Critically, cognitive dysfunction impacts negatively on the outcome of pharmacological and non-pharmacological treatments for affective disorders, where the ability to engage in and successfully complete treatment relies heavily on higher-order cognitive processes (Dunkin et al., 2000; Jaeger & Vieta, 2007). Despite knowledge of the presence of cognitive dysfunction in PTSD, to the best of our knowledge, only one study has been conducted to examine the impact of a non-standardized intervention protocol aimed at improving cognitive functioning in this population. Here, clinically (but not statistically) significant improvements were noted in a small pilot on measures of cognitive functioning after using a bottom-up executive training intervention in conjunction with transcranial direct current sample of four patients (Saunders et al., 2015).

The few studies conducted thus far with PTSD patients contrast sharply with the extant literature in schizophrenia where, to date, more than 20 randomized controlled trials in patients with psychotic-spectrum disorders have been conducted (Barlati, Deste, De Peri, Ariu, & Vita, 2013; Kurtz, 2012; Wykes, Huddy, Cellard, McGurk, & Czobor, 2011). Indeed, the results of a recent meta-analytic review point towards a moderate (effect size = 0.43) and sustained improvement in overall cognitive functioning (including for frontal-temporally mediated domains including processing speed, working memory, episodic memory, and executive functioning) among patients with schizophrenia after participating in cognitive remediation (Wykes et al., 2011). Critically, there was also a medium effect (effect size=0.37) of cognitive remediation on improving functional outcome when measured immediately post-treatment and at follow-up.

In our own laboratory, we have successfully applied cognitive remediation in patients with major depressive disorder, resulting in improvements in performance on working memory tasks in conjunction with increased functional activity in lateral prefrontal and parietal areas (Elgamel, McKinnon, Ramakrishan, Joffe, & MacQueen, 2007; Meusel, Hall, Fougere, McKinnon, & MacQueen, 2013) implicated in the CEN (Aupperle et al., 2012; Dunkin et al., 2000; Greenberg et al., 1999; Jaeger & Vieta, 2007; Johnsen & Asbjornsen, 2008; Kessler, 2000; Moradi et al., 2012; Olatunji, Cisler, & Tolin, 2007; Polak et al., 2012; Van Ameringen, Mancini, Patterson, & Boyle, 2008).

The majority of cognitive remediation interventions applied in psychiatric populations rely heavily upon a *bottom-up* restitution-based approach to training that focuses on the repair and recovery of function (Robertson & Murre, 1999). Here, participants engage in extensive, repetitive practice of tasks thought to be related to specific, cognitive domains based on the assumption that exercising memory, for example, on one task, will strengthen it for use on other memory tasks. By contrast, we are keen to investigate the efficacy of *top-down* remediation strategies that focus on the “stimulation” of higher-order systems (e.g., frontal executive systems; control of attention) to modify and regulate impaired processing in other systems (e.g., training in meta-cognitive and self-monitoring strategies). It is these interventions that we believe will have the greatest potential to restore CEN functioning. Notably, these approaches have been applied in other clinical and non-clinical populations, including older adults (Levine et al., 2007; Van Hooren et al., 2007; Winocur et al., 2007), individuals who have suffered a traumatic brain injury (Krasny-Pacini, Chevignard, & Evans, 2014; Krasny-Pacini, Limond, et al., 2014; Levine et al., 2000, 2011; Schweizer et al., 2008), have attention deficit hyperactivity disorder (ADHD) (In de Braek, Dijkstra, Ponds, & Jolles, 2012), polysubstance abuse disorder (Alfonso, Caracuel, Delgado-Pastor, & Verdejo-Garcia, 2011), or spina bifida (Stubberud, Langenbahn, Levine, Stanghelle, & Schanke, 2013), and that experience deficits in executive functioning, attention, and/or memory. Here, participants show improvement in completing everyday tasks (as measured by self-report), as well as improvements in executive functions such as decision making, working memory, and selective attention. Critically, these results are maintained at follow-up (when assessed) (Alfonso et al., 2011; In de Braek et al., 2012; Krasny-Pacini, Chevignard, et al., 2014; Krasny-Pacini, Limond, et al., 2014; Levine et al., 2000, 2007, 2011; Stubberud et al., 2013; Van Hooren et al., 2007).

Given the previous success of top-down intervention strategies in remediating frontal-, temporal- and parietally-mediated brain dysfunction across clinical populations, we hypothesize that these interventions may also have the ability to target core cognitive difficulties experienced by those suffering from PTSD. Moreover, by targeting core cognitive processes (e.g., executive functioning, decision making, and attention) mediated by frontoparietal networks involved in the CEN, we would expect to see functional reorganization of the CEN as a result of treatment (e.g., Green & Benzeval, 2013; HealthCanada, 2002; Parikh, Lam, & Group, 2001; Ratnasingham, Cairney, Rehm, Manson, & Kyndyak, 2012; World Health Organization, 2008).

## Salience network and PTSD

In addition to CEN abnormalities in PTSD, a number of studies have demonstrated connectivity alterations among brain areas related to the SN, such as between the anterior insula and other SN regions, including the amygdala (Birn, Patriat, Phillips, Germain, & Herringa, 2014; Cisler et al., 2013, 2014; Fonzo et al., 2010; Peterson, Thome, Frewen, & Lanius, 2014; Rabinak et al., 2011; Shang et al., 2014; Simmons, Norman, Spadoni, & Strigo, 2013; Simmons et al., 2008, 2011; Sripada, King, Garfinkel, et al., 2012; Sripada, King, Welsh, et al., 2012; Stevens et al., 2013; Tursich, Ros, Frewen, Calhoun, & Lanius, 2015). Fonzo et al. (Fonzo et al., 2013) also have shown specific effects of childhood maltreatment on SN functioning in PTSD during emotional face processing, where childhood maltreatment was negatively correlated with connectivity between the insula and amygdala when viewing fearful and angry faces, but positively correlated with prefrontal-limbic connectivity when viewing fearful faces. In addition, Simmons et al. (Simmons et al., 2013) found that individuals in remission from PTSD showed an increase in functional connectivity between the left ventral anterior insula and the right anterior cingulate and middle frontal gyrus, as well as with left cerebellum. Finally, Cisler et al. (Cisler et al., 2014) demonstrated that repeated exposure to a traumatic memory during the experimental paradigm increased connectivity between the right anterior insula and both the right hippocampus and right amygdala, as well as between the right posterior insula and medial prefrontal cortex, whereas patients with higher PTSD symptoms showed this effect less strongly. On balance, the results of these studies suggest that altered connectivity within the SN may result in a change in threat-sensitivity circuits, contributing to the hypervigilance and hyperarousal symptoms in PTSD. Indeed, these findings are consistent with previous models that have associated *increased* anterior insula activation with heightened levels of interoception and awareness of bodily arousal during states of emotional undermodulation, including reexperiencing and hyperarousal symptoms (Hopper, Frewen, Van der Kolk, & Lanius, 2007; Lanius, Bluhm, & Frewen, 2011; Lanius, Vermetten, et al., 2010; Paulus & Stein, 2006; Shin & Liberzon, 2010).

However, it is worth noting here that *decreased* insula activation has been associated with emotional detachment or overmodulation of emotion, hypoarousal, and attenuated interoceptive awareness of bodily arousal, such as may be involved during states of depersonalization and derealization (Hopper et al., 2007), which are accompanied by increased prefrontal inhibition of limbic regions (Lanius, Vermetten, et al., 2010). With regard to interoceptive awareness, emotional overmodulation is frequently associated with alexithymia, the inability to know what one is feeling or not having words for one's feelings, and symptoms of alexithymia have been shown to be negatively correlated with anterior insula functioning in PTSD (Frewen et al., 2008). Hence, it is possible that differential activation of the insula underlies both emotional over- and undermodulation and associated alterations in interoceptive awareness in PTSD.

## Normalizing arousal and interoceptive awareness through regulation of the SN

As described above, traumatized individuals often cycle between states of emotional undermodulation associated with hyperarousal, and emotional overmodulation associated with emotional detachment and hypoarousal, symptoms that have been associated with insula over- and underactivity, respectively (Frewen & Lanius, 2006; Hopper et al., 2007; Lanius, Vermetten et al., 2010). Traumatized individuals who exhibit emotional overmodulation frequently suffer from a profound detachment from their emotional states and a lack of interoceptive awareness as evidenced by dissociative experiences such as depersonalization and derealization (Lanius et al., 2011; Lanius, Frewen, Vermetten, & Yehuda, 2010), emotional numbing (Krystal, 1968; Krystal & Krystal, 1988; Van der Kolk & McFarlane, 1996), and alexithymia (Badura, 2003; Frewen et al., 2008; Yehuda et al., 1997), with accompanying insula underactivity. Detachment from one's emotional states often occurs in response to repeated traumatization during which the traumatized individual is frequently unable to initiate defensive actions due to overwhelming feelings and emotions. Emotional shutdown, numbness, and detachment instead ensue to the point where the traumatized person can become devoid of *any* positive (Etter, Gauthier, McDade-Montez, Cloitre, & Carlson, 2013; Frewen, Dean, & Lanius, 2012; Frewen, Dozois, & Lanius, 2012) and negative (Nawijn et al., 2015) emotional experience. Critically, without emotional experience, the capacity for salience detection becomes significantly impaired as emotions are crucial to directing behavior and physiological states to the most important actions, with the goal of maintaining homeostasis. The capacity to pursue the most salient endeavors in the present is therefore often lost in the aftermath of trauma. How can we help traumatized clients to awaken from this shutdown state and restore their interoceptive awareness and salience detection, which we hypothesize would have the concomitant effect of normalizing functioning of the insula and restoring the integrity of the SN?

Body scan meditations are a core part of the mindfulness-based stress reduction program developed by Jon Kabat-Zinn (Kabat-Zinn, 1990) and are thought to enhance interoceptive awareness and assist in overcoming emotional detachment in traumatized populations (e.g., Follette, Briere, Rozelle, Hopper, & Rome, 2014; Frewen & Lanius, 2015). Body scans encourage individuals to become aware of and to monitor bodily sensations experienced throughout the body. Given that an individual's subjective conscious emotional experience is thought to be based on the perception of physical sensations/bodily states arising from musculoskeletal, autonomic nervous, and endocrine system influences (Barrett, Mesquita, Ochsner, & Gross, 2007; Damasio, 1996; James, 1884) mediated by the anterior insula (Craig, 2010; Critchley, Wiens, Rotshtein, Öhman, & Dolan, 2004), mapping what physical sensations are associated with specific emotions can then be used to help individuals identify what emotional feelings they experience. In support of this, a recent large cross-cultural study showed that specific maps of bodily sensations are associated with different emotions (Nummenmaa, Glerean, Hari, & Hietanen, 2014). Increasing awareness of bodily sensations and encouraging individuals to map what sensations are associated with specific emotions may therefore be an important strategy in overcoming emotional detachment and thus restore salience detection and insular and SN functioning in traumatized individuals. Interestingly, meditators have also been shown to exhibit greater gray matter thickness of the insula as compared to non-meditators (Holzel et al., 2008; Lazar et al., 2005), and a significant increase in right insula cortical thickness that correlated with decreased levels of alexithymia was reported in meditation naïve subjects following mindfulness-based stress reduction (Santarnecchi et al., 2014). It is also interesting to note that emerging evidence points to the efficacy of mindfulness-based interventions in the treatment of PTSD and related disorders (Bormann, Oman, Walter, & Johnson, 2014; Kearney, McDermott, Malte, Martinez, & Simpson, 2012; Kimbrough, Magyari, Langenberg, Chesney, & Berman, 2010; King et al., 2013; Niles et al., 2012). We predict that mindfulness-based treatments, specifically body scans coupled with helping individuals to identify which bodily sensations are associated with what specific emotions, would help to overcome emotional detachment and reestablish emotional awareness, salience detection, normalize insula functioning, and restore the integrity of the SN. Such interventions will therefore need to be the focus of future investigations (Koch et al., 2014) for medication (oxytocin)-enhanced psychotherapy to normalize salience processing.

In contrast to emotional overmodulation and associated symptoms of emotional detachment, emotional undermodulation and related hyperarousal symptoms are frequently associated with hypervigilance, increased levels of interoceptive awareness salience detection, and heightened insula activation. Here, we predict that increased insula activation will alter the functioning of the SN, thereby contributing to the hypervigilance and hyperarousal symptoms in PTSD. Such symptoms have been shown to be managed by anxiety management skills that are part of standard cognitive behavioral treatments, including relaxation training, breathing exercises, fostering mindful awareness, decreasing ruminations, and thought stopping to decrease distressing thoughts and associated worries. These treatments would therefore be expected to normalize interoceptive awareness and restore salience detection through normalization of insula and SN functioning. Future studies examining individuals with PTSD will need to examine the capacity for plastic modulation of the insula and the SN through specific treatments targeting hyperarousal symptoms.

## Default mode network and PTSD

As described above, the DMN has been shown to play a key role in self-referential processing, autobiographical memory, and social cognition. Resting-state and task-related studies of PTSD participants show altered connectivity within brain structures associated with the DMN (Birn et al., 2014; Bluhm et al., 2009; Chen & Etkin, 2013; Cisler et al., 2013, 2014; Fonzo et al., 2013; Jin et al., 2014; Kennis, Rademaker, Van Rooij, Kahn, & Geuze, 2015; Lanius, Bluhm, et al., 2010; Lanius et al., 2005; Peterson et al., 2014; Qin et al., 2012; Shang et al., 2014; Sripada, King, Welsh, et al., 2012; St. Jacques et al., 2013; Tursich et al., 2015; Yin et al., 2011; Zhou et al., 2012). Moreover, the strength of connectivity within the DMN appears to correlate with PTSD symptom severity in patients with PTSD or acute posttraumatic stress symptoms (Birn et al., 2014; Cisler et al., 2013; Kennis, Rademaker, Van Rooij, Kahn, & Geuze, 2013; Lanius, Bluhm, et al., 2010; Sripada, King, Welsh, et al., 2012).

In addition, there is emerging evidence that altered resting-state connectivity between key regions of the DMN and regions associated with the SN, including the amygdala and anterior insula, may be a prognostic indicator of PTSD symptomatology (Lanius, Bluhm, et al., 2010; Zhou et al., 2012). Both Lanius et al. (Lanius, Bluhm, et al., 2010) and Zhou et al. (Zhou et al., 2012) found that resting connectivity between the PCC and left and/or right amygdala shortly after a traumatic event predicted PTSD symptom severity at 12 weeks posttrauma; the directionality of this effect, however, has not been clearly identified. Interestingly, Qin et al. (Qin et al., 2012) also found that connectivity of PCC with anterior insula distinguished between people who would later develop PTSD and those who would not, although they did not find associations between amygdala connectivity and PTSD symptoms. Studies have also reported alterations in DMN and SN regions in PTSD, suggesting an alteration within the interaction between large-scale brain networks (Bluhm et al., 2009; Jin et al., 2014; Kennis et al., 2013; Simmons et al., 2011, 2013; St. Jacques et al., 2013).

## Restoring self-related processes and the sense of self through normalization of the DMN

Trauma can have lasting effects on the sense of self (Schore, 2003) manifested both cognitively and somatically. This is evidenced by altered core beliefs, disrupted self-referential processing (including poor emotional awareness, alexithymia), and dissociative symptoms including depersonalization and related identity disturbance. It is not unusual for traumatized individuals to experience altered beliefs about themselves, for example: “I have permanently changed for the worse,” “I will never be able to feel normal emotions again,” or “I don't know myself anymore” [see Posttraumatic Cognition Inventory (Foa, Ehlers, Clark, Tolin, & Orsillo, 1999) and Cox, Resnick, & Kilpatrick (2014)]. In addition to these cognitive alterations in self-referential processing, individuals with PTSD may also exhibit somatically-based alterations in self-referential processing, such as depersonalization and related identity disturbance, manifested by feelings like “I feel as if I am outside my body,” “I feel dead inside,” or “I feel like my body does not belong to me” (Bernstein & Putnam, 1986; Briere, 2002; Dell, 2006; Foa et al., 1999).

It has been suggested that the DMN is a key brain network underlying the continuous experience of the sense of self across time and into the future, given its role in autobiographical memory retrieval, envisioning the future, and conceiving the perspectives of others; these are all processes that have been shown to be disrupted in PTSD (Brewin, 2014; Brown et al., 2013, 2014; Freeman, Hart, Kimbrell, & Ross, 2009; Mazza et al., 2012; Moore & Zoellner, 2007; Nazarov, Frewen, Oremus, et al., 2014; Nazarov, Frewen, Parlar, et al., 2014; St. Jacques et al., 2013). Moreover, dissociative experiences, including depersonalization, have repeatedly also been shown to involve brain regions associated with the DMN, including the medial prefrontal cortex, medial parietal lobe, and the temporoparietal junction (reviewed in (Frewen & Lanius, 2015)), which suggests that alterations in the DMN are also a potential mechanism underlying depersonalization and related identity disturbances that follow psychological trauma. We would therefore expect that current PTSD treatments geared towards alleviating both cognitive and somatically-based disturbances in self-referential processing (including, but not limited to, cognitive processing therapy, exposure therapy, eye movement desensitization and reprocessing, mentalization based therapy, dialectical behavior therapy, trauma affect regulation: guide for education and therapy, emotion focused therapy, skills training in affective and interpersonal regulation, psychodynamic approaches, and sensorimotor) may aid in the restoration of DMN function and in reestablishing sense of self. As such, it is critical that future research explicitly measures changes in the sense of self as a treatment outcome. This effort will necessitate the development of appropriate measures to capture this complex concept. In addition, investigations examining whether a combination of cognitive and body-focused treatments, rather than each of these treatments alone, can more optimally relieve self-dysfunction and restore DMN integrity, will be necessary. In theory, combination treatment may simultaneously target both cognitive and somatically-based self-dysfunction through top-down and bottom-up processing, respectively.

## Neurofeedback as a tool to aid in the restoration of large-scale brain network functioning

Neurofeedback is a form of biofeedback that uses a brain computer interface to provide feedback about brain functioning in the form of an electroencephalogram (EEG) or blood oxygenation level dependent response, thereby enabling self-regulation of brain activity. EEG neurofeedback training has been shown to aid in the regulation of major brain networks such as the SN and the DMN. For example, a recent functional magnetic resonance imaging (fMRI) study examined the effects of a single session of neurofeedback training versus SHAM (placebo neurofeedback) aimed at voluntarily reducing the alpha rhythm amplitude to gain insight into potential mechanisms underlying this form of neurofeedback. Activity in the alpha band (8–12 Hz) has previously been shown to be successfully modulated by naïve participants through neurofeedback (NFB) (Ros, Munneke, Ruge, Gruzelier, & Rothwell, 2010), and evidence suggests that alpha rhythm NFB may be beneficial in treating anxiety and attention problems (Hardt & Kamiya, 1978; Rasey, Lubar, McIntyre, Zoffuto, & Abbott, 1995), symptoms relevant for the treatment of PTSD and related disorders. In healthy individuals, alpha neurofeedback training led to plastic modulation of both SN and DMN functional connectivity, effects which were not observed in the SHAM condition (Ros et al., 2013).

A similar investigation was subsequently conducted in PTSD patients who had suffered repeated childhood trauma. The results of this study indicated that voluntarily reducing alpha rhythm amplitude was associated with decreased alpha amplitude during training, followed by a significant increase (“rebound”) in resting-state alpha rhythm amplitude. This rebound was related to increased calmness, greater default mode network connectivity, and enhanced SN connectivity (Kluetsch et al., 2014). The alpha “rebound,” which was only observed in the PTSD patients and not in healthy controls, may represent a form of metaplasticity (Muller-Dahlhaus & Ziemann, 2014). Metaplasticity is thought to allow the brain to maintain homeostasis, thus keeping network activity within a physiological range. Hence, the alpha rebound could be seen as a reaction of the brain towards restoring alpha activity to an optimal level for cognitive function (Ros, Baars, Lanius, & Vuilleumier, 2014). Although studies examining mechanisms underlying alpha neurofeedback training in healthy individuals and persons suffering from PTSD need to be repeated following multiple neurofeedback training sessions, they nevertheless point to promising effects this intervention may have for regulating key brain networks, including the SN and DMN.

Although, to our knowledge, no studies have examined the use of real-time fMRI in the regulation of large-scale networks in PTSD, studies in both healthy controls (Lawrence et al., 2013; Paret et al., 2014; Zhang, Yao, Zhang, Long, & Zhao, 2013; Zotev, Phillips, Young, Drevets, & Bodurka, 2013), meditators (Garrison, Santoyo, et al., 2013; Garrison, Scheinost, et al., 2013), and certain psychiatric disorders, including schizophrenia (Ruiz et al., 2013) and depression (Linden et al., 2012; Young et al., 2014; Yuan et al., 2014; Zotev, Phillips, Yuan, Misaki, & Bodurka, 2014), reveal promising results for the plastic modulation of brain regions such as the insula, amygdala, and PCC involved in the SN and DMN as a function of real-time fMRI neurofeedback. Future studies will need to examine this type of intervention for PTSD and related symptoms.

Another neural treatment, repetitive transmagnetic stimulation (rTMS), involves non-invasive, superficial stimulation of the brain and has shown encouraging outcomes for the treatment of PTSD when applied to right or left dorsolateral prefrontal cortex (for meta-analysis see (Berlim & Van den Eynde, 2014)). However, no studies have yet examined the effects of rTMS on the integrity of large-scale brain networks in PTSD, and future investigations of this type may also provide insight into the brain mechanisms underlying successful treatment. Finally, novel pharmacotherapeutic treatments (Watts et al., 2013) developed specifically to target intrinsic network functioning in PTSD would provide important research opportunities.

## Concluding remarks

**Fig. 1:**
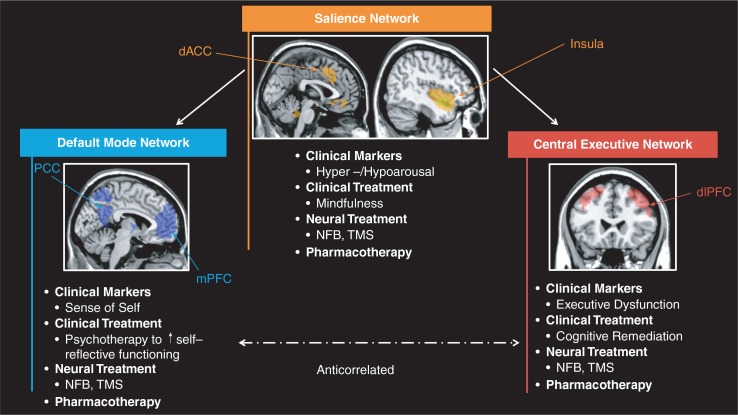
Neuroscientifically-informed treatment interventions in psychotraumatology: Three intrinsic networks, including the central executive network (CEN), salience network (SN), and default mode network (DMN) may be associated with specific clinical symptoms observed in PTSD, including cognitive dysfunction (CEN), hyper- and hypoarousal/interoception (SN), and an altered sense of self (DMN). Specific testable treatment interventions targeted to restore each one of these brain networks and related clinical dysfunction are suggested. Images were created using network templates available from http://findlab.stanford.edu/functional_ROIs.html (Shirer, Ryali, Rykhlevskaia, Menon, & Greicius, 2012).

This commentary has demonstrated increasing evidence for altered functioning of three large-scale brain networks in PTSD, namely the CEN, SN, and DMN. We propose that each network is associated with specific clinical symptoms observed in PTSD, including cognitive dysfunction (CEN), hyper- and hypoarousal/interoception (SN), and an altered sense of self (DMN). Specific testable treatment interventions targeted to restore each of these neural networks and related clinical dysfunction were suggested (Fig. 1). Neuroscientifically-informed integrative treatment interventions will be central to research efforts aimed at targeting specific PTSD and related symptoms.
